# Multi-Target State Extraction for the SMC-PHD Filter

**DOI:** 10.3390/s16060901

**Published:** 2016-06-17

**Authors:** Weijian Si, Liwei Wang, Zhiyu Qu

**Affiliations:** College of Information and Communication Engineering, Harbin Engineering University, Harbin 150001, China; swj0418@263.net (W.S.); wang080006@hrbeu.edu.cn (L.W.)

**Keywords:** multi-target tracking, probability hypothesis density filter, state extraction

## Abstract

The sequential Monte Carlo probability hypothesis density (SMC-PHD) filter has been demonstrated to be a favorable method for multi-target tracking. However, the time-varying target states need to be extracted from the particle approximation of the posterior PHD, which is difficult to implement due to the unknown relations between the large amount of particles and the PHD peaks representing potential target locations. To address this problem, a novel multi-target state extraction algorithm is proposed in this paper. By exploiting the information of measurements and particle likelihoods in the filtering stage, we propose a validation mechanism which aims at selecting effective measurements and particles corresponding to detected targets. Subsequently, the state estimates of the detected and undetected targets are performed separately: the former are obtained from the particle clusters directed by effective measurements, while the latter are obtained from the particles corresponding to undetected targets via clustering method. Simulation results demonstrate that the proposed method yields better estimation accuracy and reliability compared to existing methods.

## 1. Introduction

Multi-target tracking (MTT) has to deal with the detection and estimation problems of multiple targets in a cluttered environment [1]. Traditional solutions such as multiple hypothesis tracking (MHT) filter and joint probabilistic data association (JPDA) filter handle this problem through a divide-and-conquer approach that involves data association and filtering processes [2,3]. As an alternative to the association-based methods, the random finite sets (RFS) approach is an emerging technique to multi-target tracking (MTT), and the resulting optimal multi-target Bayes filter provides a rigorous theoretical basis for many novel multi-target filters [4,5,6]. In this context, the probability hypothesis density (PHD) filter [4] which is derived via first-order moment approximation of the multi-target posterior density, and its implementations such as sequential Monte Carlo PHD (SMC-PHD) filter [7,8] and Gaussian mixture PHD (GM-PHD) filter [9], have been widely studied in the area of MTT over the last decade [10,11,12,13,14].

From an engineering point of view, the SMC-PHD filter is more suitable for practical applications due to its ability to accommodate both nonlinear and non-Gaussian dynamics [8,13]. However, the SMC method leads to a troublesome problem in extracting the estimates of target states from the given particle approximation of the PHD (also known as intensity function), and the accuracy of the estimated multi-target state directly determines the tracking performance of a multi-target filtering algorithm. Thus it is a critical issue for the SMC-PHD filter to develop a reliable multi-target state extraction algorithm, which has attracted significant attention [15,16,17,18,19,20,21]. Typically, the clustering techniques such as k-means clustering [7,15] and c-means fuzzy clustering [16], and finite mixture models (FMM) algorithm via expectation-maximization (EM) [15] or Markov chain Monte Carlo (MCMC) sampling [17], have been investigated in this aspect. The results in [15] demonstrated that the k-means clustering outperforms the FMM algorithm with potential computational efficiency and fewer spurious estimates. In addition, the CLEAN method [18] was proposed to extract target states from the SMC-PHD filter. However, since this method only exploits the weight information of particles, the average performance of the CLEAN method is no better than the *k*-means clustering in general. Subsequently, the method in [19] introduced clustering algorithms to overcome the drawbacks of the CLEAN technique. Despite this, the effect is limited because the clustering algorithms [15,16] are particularly problematic and unreliable due to the hard limiting on the number of clusters (specified by the estimated number of targets), especially in the scenarios where there exist closely spaced targets or incorrect estimates of target number [19,20,21]. Moreover, such methods have high computational cost because of the requirement of iterative computing process.

To address these problems, some measurement-oriented methods that consider the relationships between weighted particles and measurements have been proposed to perform state extraction [20,21], where the *ad-hoc* particle clustering methods mentioned above are eliminated. Typically, the grouping method [20], implemented by replicating the current particle set and re-weighting them corresponding to individual measurements, has been a popular state extraction method in different versions of the SMC-PHD filter [10,12]. In contrast, the method in [21] introduced a maximum likelihood criterion for particle clustering. More recently, a systematic and theoretical analysis about the possibility of extracting point estimates from PHD with respect to the optimal sub-pattern assignment (OSPA) [22] metric was investigated in [23], however, how to design a reasonable loss function and extend the method to general cases need to be further researched. To facilitate the parallel implementation of the SMC-PHD filter, a new multi-expected a posterior (MEAP) method was outlined in [24], where the estimation procedure can still be seen as a kind of measurement-oriented technique by introducing particle-to-measurement association based on the near and nearest neighbor principle. Moreover, the concept of association measure was introduced to the PHD recursion [25], which theoretically makes it possible to extract track estimates from the PHD filter. Unfortunately, due to the complexity of measurement association and the fast augmentation of the number of observation paths, the feasible implementation method and its potential performance are not clear. Generally speaking, the measurement-oriented methods are more acceptable and practical than the clustering-based methods in terms of both reliability and computational efficiency [20,24]. On the other hand, the existing solutions of this category are all limited by the fact that only the targets that have been detected may have chances to be reported, which will result in inaccurate estimation when missed detections occur. Since the PHD recursion is sensitive to missed detections, to date, how to extract state estimates from the SMC-PHD filter accurately in the presence of detection uncertainty still remains a challenge.

The key contribution of this paper is a novel solution to achieving multi-target state extraction in the SMC-PHD filter. More specifically, the normalized likelihoods of the predicted particles with respect to individual measurements are introduced to develop a validation mechanism which aims at selecting effective measurements and particles, *i.e.*, the target-originated measurements and the particles corresponding to detected targets. Subsequently, by constructing the association probability distributions between particles and measurements, the particles of detected targets are divided into different clusters corresponding to the effective measurements in a probabilistic manner. Then, according to the estimated target number, the point estimates of the target locations can be extracted from the resulting clusters. Moreover, benefiting from the proposed validation mechanism, we introduce a gating technique to further identify the particles of undetected targets, and then extract the corresponding target states, thereby implying an improved estimation performance in the circumstances with detection uncertainty. Simulation results demonstrate the effectiveness of our methods in comparison with the existing methods.

The remainder of this paper is organized as follows: Section 2 provides a brief review of the PHD filter and the SMC-PHD filter. The proposed multi-target state extraction method is presented in Section 3. Simulation results and analysis are presented in Section 4 and conclusions are drawn in Section 5.

## 2. Problem Formulation

### 2.1. The PHD Filter

The RFS method provides an elegant representation of multi-target systems. For example, let *n_k_* and *m_k_* represent the time-varying number of targets and measurements at time *k*, respectively, then the corresponding target states ***x****_k_*_,1_,***x****_k_*_,2_,…***x****_k,nk_* and measurements ***z****_k_*_,1_,***z****_k_*_,2_,…***z****_k,mk_* can naturally be modeled as the finite sets *X_k_* = {***x****_k_*_,1_,***x****_k_*_,2_,…***x****_k,nk_*} and *Z_k_* = {***z****_k_*_,1_,***z****_k_*_,2_,…***z****_k,mk_*}, respectively [7,8]. Based on the RFS theory, the optimal multi-target Bayes filter was developed by Mahler. However, the calculations of high-dimensional integration of multi-target densities make it intractable to implement the full multi-target Bayes filter directly. To obtain a practical solution, the sub-optimal PHD filter was derived via moment approximation [4], which only recursively propagates the posterior intensity of the multi-target RFS in time. Under the assumptions of Poisson multi-target distributions [4], the PHD filter (without considering target spawning) is given by:

(1) $$v_{k|k-1}(x)=\int p_{S,k}(\zeta )f_{k|k-1}(x|\zeta )v_{k-1}(\zeta )d\zeta +\;\gamma_{k}(x)$$

(2) $$v_{k}(x)=[1-p_{D,k}(x)]v_{k|k-1}(x)+\sum_{z\in Z_{k}}\frac{p_{D,k}(x)g_{k}(z|x)v_{k|k-1}(x)\;}{\kappa_{k}(z)+\int p_{D,k}(\zeta )g_{k}(z|\zeta )v_{k|k-1}(\zeta )d\zeta }$$

here $v_{k}(\cdot )$ is the posterior intensity associated with the multi-target state at time k, $\gamma_{k}(\cdot )$ is the intensity of newborn targets, $p_{S,k}(\zeta )$ is the probability that a target will survive at time k given the state ζ at the previous time step. $f_{k|k-1}(\cdot |\zeta )$ is the transition probability density of a single target, $p_{D,k}(x)$ is the probability of detection for a target with state x, $g_{k}(z|\cdot )$ is the measurement likelihood of individual targets, and $\kappa_{k}(z)$ is the intensity of clutter. The integral of the PHD over the state space is the expected number of targets and the peaks of the PHD can be used to generate target state estimates [7].

### 2.2. Review of the SMC-PHD Filter

Although the PHD filter alleviates the computational complexity of the multi-target Bayes filter to a great extent, it can be seen from Equations (1) and (2) that the PHD recursion still involves multiple integrals. The implementation of the PHD filter generally resorts to some approximate methods, and we focus on the SMC implementation proposed in [7], henceforth referred to as the SMC-PHD filter. Suppose that at time k=0, $L_{0}$ particles ${\left\{x_{0}^{\left(i\right)}\right\}}_{i=1}^{L_{0}}$ are drawn for the prior PHD and their weights are assigned as ${\left\{w_{0}^{\left(i\right)}\right\}}_{i=1}^{L_{0}}$, where $x_{0}^{\left(i\right)}$ is the *i*th state particle and $w_{0}^{\left(i\right)}$ is the associated weight. The main steps of the SMC-PHD filter at time k>0 are briefly summarized as follows:

*Step 1 Prediction*:
  - For $\;i=1,...,L_{k-1}\;$, sample ${\tilde{x}}_{k}^{\left(i\right)}$ from a proposal density $q_{k}(\cdot |x_{k-1}^{\left(i\right)},Z_{k})$ for persistent targets and compute the predicted weights:
    (3) $${\tilde{w}}_{k|k-1}^{\left(i\right)}=\frac{\phi_{k|k-1}({\tilde{x}}_{k}^{\left(i\right)},x_{k-1}^{\left(i\right)})w_{k-1}^{\left(i\right)}}{q_{k}({\tilde{x}}_{k}^{\left(i\right)}|x_{k-1}^{\left(i\right)},Z_{k})}$$
    where $\phi_{k|k-1}({\tilde{x}}_{k}^{\left(i\right)},x_{k-1}^{\left(i\right)})=p_{S,k}(x_{k-1}^{\left(i\right)})f_{k|k-1}({\tilde{x}}_{k}^{\left(i\right)}|x_{k-1}^{\left(i\right)})$.
  - For $i=L_{k-1}+1,...,L_{k-1}+J_{k}$, sample ${\tilde{x}}_{k}^{\left(i\right)}$ from a proposal density $p_{k}(\cdot |Z_{k})\;$ for newborn targets and compute the corresponding weights:
    (4) $${\tilde{w}}_{k|k-1}^{\left(i\right)}=\frac{\gamma_{k}({\tilde{x}}_{k}^{\left(i\right)})}{J_{k}p_{k}({\tilde{x}}_{k}^{\left(i\right)}|Z_{k})}$$
    where $J_{k}$ is the number of particles for newborn targets.
*Step 2 Update*:
  - For each $z\in Z_{k}$, compute:
    (5) $$C_{k}(z)=\sum_{j=1}^{L_{k-1}+J_{k}}\psi_{k,z}({\tilde{x}}_{k}^{\left(j\right)}){\tilde{w}}_{k|k-1}^{\left(j\right)}$$
    where $\psi_{k,z}({\tilde{x}}_{k}^{\left(j\right)})=p_{D,k}({\tilde{x}}_{k}^{\left(j\right)})g_{k}(z|{\tilde{x}}_{k}^{\left(j\right)})$ and $g_{k}(z|{\tilde{x}}_{k}^{\left(j\right)})$ is the likelihood of a measurement z resulting from a particle ${\tilde{x}}_{k}^{\left(j\right)}$.
  - For $i=1,...,L_{k-1}+J_{k}$, update weights:
    (6) $${\tilde{w}}_{k}^{\left(i\right)}=\left[(1-p_{D,k}({\tilde{x}}_{k}^{\left(i\right)}))+\sum_{z\in Z_{k}}\frac{\psi_{k,z}({\tilde{x}}_{k}^{\left(i\right)})\;}{\kappa_{k}(z)+C_{k}(z)}\right]{\tilde{w}}_{k|k-1}^{\left(i\right)}$$
*Step 3 Resampling*:
  - Compute the total mass ${\hat{N}}_{k|k}=\sum_{i=1}^{L_{k-1}+J_{k}}{\tilde{w}}_{k}^{\left(i\right)}$ and then resample ${\left\{{\tilde{w}}_{k}^{\left(i\right)}/{\hat{N}}_{k|k},{\tilde{x}}_{k}^{\left(i\right)}\right\}}_{i=1}^{L_{k-1}+J_{k}}$ to get ${\left\{w_{k}^{\left(i\right)}/{\hat{N}}_{k|k},x_{k}^{\left(i\right)}\right\}}_{i=1}^{L_{k}}$.
  - Rescale the weights by ${\hat{N}}_{k|k}$ to obtain ${\left\{w_{k}^{\left(i\right)},x_{k}^{\left(i\right)}\right\}}_{i=1}^{L_{k}^{}}$.
*Step 4 Multi-Target Parameter Estimation*:
  - Estimate the number of targets $N_{k}$ (by rounding ${\hat{N}}_{k|k}$).
  - Extract the target state set ${\hat{X}}_{k}=\{{\hat{x}}_{k}^{\left(1\right)},...,{\hat{x}}_{k}^{\left(N_{k}\right)}\}$ from the particles that represent the posterior intensity, where ${\hat{x}}_{k}^{\left(1\right)},...,\;{\hat{x}}_{k}^{\left(N_{k}\right)}$ denote the estimated multi-target state.

More details on the mathematical derivation and analysis can be found in [7]. Note that in standard SMC-PHD filter, the process of state estimation is not a necessary step and has no influence on the filter recursion itself, but for the purpose of MTT where the number and instantaneous positions of all targets need to be estimated, we consider this process as *Step 4* in the original algorithm. The main contribution of our study is the techniques for Step 4 of the SMC-PHD filter and more specifically on the extraction of individual target states.

## 3. The Proposed Multi-Target State Extraction Method

According to the definition of PHD [4], its peaks in the state space indicate the points with the largest expected target density. The SMC-PHD filter propagates a set of weighted particles to approximate the posterior intensity associated with the multi-target density, and the weights of different particles characterize the intensities at exactly the locations of the corresponding state particles [7]. During the filtering iteration, all measurements in $Z_{k}\;$ including both target-originated measurements and clutter are used to update the weights of particles. Besides, some targets may not be detected due to detection uncertainty. Therefore, it is preferable to identify the measurements and particles that are closely related and decide whether they are related to the existing targets in a principled manner, which in turn can be exploited to provide accurate and reliable state estimation.

### 3.1. Particles and Measurements Classification

Since no explicit associations between measurements and targets are required in the SMC-PHD filter, all measurements are used to update the weight of each particle. A close inspection of Equation (6) reveals that the updated weight ${\tilde{w}}_{k}^{\left(i\right)}$ can be decomposed into a series of individual weight components corresponding to $z_{k,m}\in Z_{k}\;(m=1,2,...,m_{k})$, which can be written as:

(7) $$w_{k}^{i}(z_{k,m})=\left\{ \begin{array}{ll} (1-p_{D,k}({\tilde{x}}_{k}^{\left(i\right)})){\tilde{w}}_{k|k-1}^{\left(i\right)} & z_{k,m}=\emptyset \;\\ \frac{\psi_{k,z_{k,m}}({\tilde{x}}_{k}^{\left(i\right)})\;}{\kappa_{k}(z_{k,m})+C_{k}(z_{k,m})}w_{k|k-1}^{\left(i\right)} & z_{k,m}\in Z_{k} \end{array}\right.$$

where $z_{k,m}=\emptyset$ represents the term caused by missed detection, and:

(8) $${\tilde{w}}_{k}^{\left(i\right)}=\sum_{z\in \{\emptyset ,Z_{k}\}}w_{k}^{i}(z)$$

According to the representation in Equation (7), for a detected target represented by particle ${\tilde{x}}_{k}^{\left(i\right)}$, the weight component $w_{k}^{i}(z_{k,m})$ measures the contribution degree that $z_{k,m}$ makes to the updated weight of ${\tilde{x}}_{k}^{\left(i\right)}$, which is essentially established by the likelihood of the particle with respect to the underlying measurement. Based on this fact, the particles’ normalized likelihood distribution corresponding to measurement $z_{k,m}$ can be defined as:

(9) $$p_{k}^{m,i}=\frac{g_{k}(z_{k,m}|{\tilde{x}}_{k}^{\left(i\right)})}{\kappa_{k}(z_{k,m})+\sum_{i=1}^{L_{k-1}+J_{k}}g_{k}(z_{k,m}|{\tilde{x}}_{k}^{\left(i\right)})}\;,\;i=1,...,L_{k-1}+J_{k}$$

where $\kappa_{k}(z_{k,m})$ is introduced to suppress the influence of clutter. Otherwise, when $z_{k,m}$ is a clutter measurement that results in non-zero likelihoods for some particles, no matter how small these values are, there will be some large $p_{k}^{m,i}$ after the likelihood normalization processes. It is clear that $0\leq p_{k}^{m,i}\leq 1$. To identify the particles of detected targets, the following criterion is defined under the premise that there must be true measurements among $Z_{k}$.

*Criterion 1*:  The particles matching with a certain measurement in terms of significant likelihoods are more likely to represent detected targets.

Thus, given the measurement set $Z_{k}\;$, the particle ${\tilde{x}}_{k}^{\left(i\right)}$ that satisfies:

(10) $$\;\sum_{m=1}^{m_{k}}\text{Logic}(p_{k}^{m,i}\geq \gamma_{g})>0$$

is selected as a candidate particle for detected targets, where Logic(⋅) is defined as a logic operation with Logic(1)=1 and Logic(0)=0, $\gamma_{g}$ is a preset threshold. An intuitive interpretation of Equation (10) is that the particle associated with at least one measurement with significant normalized likelihood is regarded as a candidate particle for detected targets. Note that when two or more measurements are closely spaced, the one-to-many relationship may occur. Repeating the process of Equation (10) for all ${\left\{{\tilde{x}}_{k}^{\left(i\right)}\right\}}_{i=1}^{L_{k-1}+J_{k}}$, and then all the resulting candidate particles are collected to construct a new weighted particle set:

(11) $$X_{C,k}={\left\{w_{k|k-1}^{\left(i^{\prime }\right)},{\tilde{x}}_{k}^{\left(i^{\prime }\right)}\right\}}_{i^{\prime }=1}^{N_{c}}$$

where $N_{c}$ is the total number of the candidate particles and $i^{\prime }$ is the new index of the particle in $X_{C,k}$. As a result, the potential particles for undetected targets are given by:

(12) $${\bar{X}}_{k}={\left\{w_{k|k-1}^{\left(i\right)},{\tilde{x}}_{k}^{\left(i\right)}\right\}}_{i=1}^{L_{k-1}+J_{k}}-{\left\{w_{k|k-1}^{\left(i^{\prime }\right)},{\tilde{x}}_{k}^{\left(i^{\prime }\right)}\right\}}_{i^{\prime }=1}^{N_{c}}$$

On the other hand, Equation (8) further reveals that although all measurements are considered to update the particle weight, only the true measurement for the particle will generate a large weight component that represents a potential target in the state space. Thus, another validation technique is proposed to determine whether a measurement $z_{k,m}\in Z_{k}$ is effective by:

(13) $$\sum_{i^{\prime }=1}^{N_{c}}\text{Logic}(p_{k}^{m,i^{\prime }}\geq \gamma_{g})\geq \tau_{n}$$

where $\tau_{n}$ denotes a threshold of the acceptable number of particles associated with a potential true measurement. The implication is that Equation (13) confirms the effectiveness of different measurements based on the candidate particles in $X_{C,k}$, which is mutually complementary with Equation (10). Therefore, it is straightforward to obtain the effective measurement set:

(14) $$Z_{E,k}={\left\{z_{k,m^{\prime }}\right\}}_{m^{\prime }=1}^{M_{e}}$$

where $M_{e}$ is the number of effective measurements. According to the principle of the SMC-PHD filter, the particles in $X_{C,k}$ can effectively capture the regions where the PHD peaks may appear after filtering update. Furthermore, $Z_{E,k}$ limits the size of the measurement set which originates from true targets with high probability and also, to a certain extent, obviates unnecessary computation caused by partitioning particles corresponding to clutter measurements.

### 3.2. Multi-Target State Extraction

For detected targets, following the basic idea behind the measurement-oriented methods, the measurements in $Z_{E,k}$ can be used to divide particles into different clusters; each one represents a possible peak in the particle approximation of the posterior intensity. It is worth emphasizing that although the idea has been adopted previously [20,21,24], the implementation presented here has significant differences. Firstly, we introduce a validation mechanism before this process, only the resulting measurement set $Z_{E,k}$ and particle set $X_{C,k}$ are considered. Secondly, the particle-to-measurement associations are established in a principled probabilistic manner, where the association probability distribution over the elements of $Z_{E,k}$ for each particle ${\tilde{x}}_{k}^{\left(i^{\prime }\right)}\in X_{C,k}$ is formulated by:

(15) $$P_{k}(m^{\prime },i^{\prime })=\frac{p_{k}^{m^{\prime },i^{\prime }}}{\sum_{m^{\prime }=1}^{M_{e}}p_{k}^{m^{\prime },i^{\prime }}}\;m^{\prime }=1,...,M_{e}$$

Then, a maximum a posterior (MAP) rule is exploited to determine the label of a particle:

(16) $$l({\tilde{x}}_{k}^{\left(i^{\prime }\right)})=\arg\;\underset{m^{\prime }=1,...,M_{e}}{\max}P_{k}(m^{\prime },i^{\prime })$$

Obviously, $l({\tilde{x}}_{k}^{\left(i^{\prime }\right)})$ is specified by one of the index $m^{\prime }$ of a measurement in $Z_{E,k}$. In addition, the weight of each particle ${\tilde{x}}_{k}^{\left(i^{\prime }\right)}$ is given by:

(17) $$w_{k}^{i^{\prime }}(z_{k,l_{i^{\prime }}})=\frac{\psi_{k,z_{k,l_{i^{\prime }}}}({\tilde{x}}_{k}^{\left(i^{\prime }\right)})\;}{\kappa_{k}(z_{k,l_{i^{\prime }}})+C_{k}(z_{k,l_{i^{\prime }}})}w_{k|k-1}^{\left(i^{\prime }\right)}$$

where the abbreviation $l_{i^{\prime }}\overset{abbr}{=}l({\tilde{x}}_{k}^{\left(i^{\prime }\right)})$ is used for notational convenience.

Based on the results of Equations (16) and (17), the main steps of the proposed method are described in Algorithm 1. First, the process of partitioning particles (in lines 1 to 3 of Algorithm 1) into different clusters determines which particle belongs to which measurement, and each cluster corresponds to a potential target. Sequentially, we extract individual target states from the $N_{k}$ clusters with the largest total weights. It can be expected that the clusters restricted by the candidate particles together with effective measurements will be more effective and accurate to capture the peaks in the PHD. Finally, the weighted mean of state particles in each cluster is taken as the estimated location. Besides, given a state estimate ${\hat{x}}_{k}^{\left(t\right)}$ for a cluster $C_{k}^{t}$, its covariance estimate ${\hat{P}}_{k}^{\left(t\right)}$ can be approximated by:

(18) $${\hat{P}}_{k}^{\left(t\right)}=\sum_{q=1}^{\left|C_{k}^{t}\right|}w_{k}^{q,t}({\tilde{x}}_{k}^{\left(q\right)}-{\hat{x}}_{k}^{\left(t\right)}){\left({\tilde{x}}_{k}^{\left(q\right)}-{\hat{x}}_{k}^{\left(t\right)}\right)}^{T}$$

where $\left|C_{k}^{t}\right|$ denotes the number of elements in $C_{k}^{t}$, ${\tilde{x}}_{k}^{\left(q\right)}$ and $w_{k}^{q,t}$ denotes the particle and its associated weight with index q in $C_{k}^{t}$.

**Algorithm 1.** Multi-target state extraction for detected targets.**Given**: The estimated target number $N_{k}$, $Z_{E,k}$ and $X_{C,k}$.Compute the label $l({\tilde{x}}_{k}^{\left(i^{\prime }\right)})$ for each particle ${\tilde{x}}_{k}^{\left(i^{\prime }\right)}$ using (16);Compute the weight of each particle using (17);Partition the new weighted particle set ${\left\{w_{k}^{i^{\prime }}(z_{k,l_{i^{\prime }}}),{\tilde{x}}_{k}^{\left(i^{\prime }\right)}\right\}}_{i^{\prime }=1}^{N_{c}}$ into $M_{e}$ clusters according to the label of each particle;Compute the weight summation $W_{k,j}$ for each nonempty cluster with index $j\in (1,2,...,M_{e})$, otherwise $W_{k,j}=0$;Choose $N_{k}$ clusters, denoted as ${\left\{C_{k}^{t}\right\}}_{t=1}^{N_{k}}$, with the largest total weights as the valid clusters;Extract the estimated state set ${\hat{X}}_{k}$ by the following two steps:
Compute the normalized particle weights in each cluster;The estimated locations are obtained by taking the weighted mean of the particle states from ${\left\{C_{k}^{t}\right\}}_{t=1}^{N_{k}}$.

Theoretically, the SMC-PHD filter handles missed detection with a special term $(1-p_{D,k}({\tilde{x}}_{k}^{\left(i\right)})){\tilde{w}}_{k|k-1}^{\left(i\right)}$, and thus preserves the information of undetected targets. However, in reality, when missed detection occurs, the SMC-PHD filter tends to give an incorrect estimation of the target numbers [26]. In this case, it is impossible to extract the estimates of the undetected targets based on measurements.

Benefiting from the particles classification method in Section 3.1, this problem can be solved by the proposed Algorithm 2. The core idea behind this method is to identify the particles corresponding to undetected targets and then extract corresponding target states. Recall that the set ${\bar{X}}_{k}$ contains all the particles that have no closely related measurements, which in turn can be interpreted as the particles corresponding to potentially undetected targets. In view of the fact that the particle distribution in the state space consists of different particle clusters which indicate the positions of persistent targets, once the set ${\hat{X}}_{k}=\{{\hat{x}}_{k}^{\left(1\right)},...,{\hat{x}}_{k}^{\left(N_{k}\right)}\}$ of estimated target states is obtained, all particles near the regions of the estimated target locations are more likely to have the identical sources. Therefore, the Mahalanobis distance is introduced to form validation gates to eliminate such particles in ${\bar{X}}_{k}$ (lines 2 to 5 of Algorithm 2), and λ is a gating threshold which is used to determine the size of the validation region for each estimated target state. Based on the dimension of the state vector and an expected gate probability $p_{g}$, λ can be obtained from the formulas in [27] (p. 337). In general, to compensate the possible statistical error in ${\hat{P}}_{k}^{\left(i\right)}$, a large gate probability which is very close to 1 is suggested to ensure overall effectiveness. Besides, the particles corresponding to newborn targets at current time should be removed (line 6 of Algorithm 2). Finally, $U_{k}$ is taken as the particle set of undetected targets.

Let ${\left\{w_{k|k-1}^{\left(p^{\prime }\right)},{\tilde{x}}_{k}^{\left(p^{\prime }\right)}\right\}}_{p^{\prime }=1}^{\left|U_{k}\right|}$ represent the weighted particles in $U_{k}$, in order to extract the estimates of the undetected targets, we first guess the number of undetected targets by:

(19) $${\bar{N}}_{U,k}=\text{round}\left(\sum_{p^{\prime }=1}^{\left|U_{k}\right|}{\tilde{w}}_{k|k-1}^{\left(p^{\prime }\right)}\right)$$

The nonzero value of ${\bar{N}}_{U,k}$ indicates that the particles in $U_{k}$ are sufficient to represent potential targets. Since no measurements are available to generate reliable weights for these particles at this stage, we suggest using the particles’ spatial distribution characteristic to extract potential target states. Thus the commonly used k-means clustering method [15] can be used to obtain the state estimate set ${\hat{X}}_{U,k}$. As a result, the modified estimation results are given by:

(20) $${\hat{X}}_{k,mod}={\hat{X}}_{k}\cup {\hat{X}}_{U,k}$$

(21) $$N_{k,mod}=N_{k}+{\bar{N}}_{U,k}$$

**Algorithm 2.** Methods for state estimation of undetected targets.**Given**: ${\bar{X}}_{k}$ and ${\hat{X}}_{k}$1. Set $U_{k}={\bar{X}}_{k}$, ℐ=∅2. **for**
$i=1,...,N_{k}$
**do**3. $ℐ=\{{\tilde{x}}_{k}^{\left(p\right)}\in U_{k}|({\tilde{x}}_{k}^{\left(p\right)}-{\hat{x}}_{k}^{\left(i\right)}){\left[{\hat{P}}_{k}^{\left(i\right)}\right]}^{-1}{\left({\tilde{x}}_{k}^{\left(p\right)}-{\hat{x}}_{k}^{\left(i\right)}\right)}^{T}\leq \lambda \}$4. $U_{k}=U_{k}\backslash ℐ$5. **end for**6. $U_{k}=U_{k}\backslash (U_{k}\cap {\left\{{\tilde{x}}_{k}^{\left(i\right)}\right\}}_{i=L_{k-1}+1}^{L_{k-1}+J_{k}})$.7. Compute ${\bar{N}}_{U,k}$ according to (19)8. **if**
${\bar{N}}_{U,k}\geq 1$9. State extraction using clustering method10. **end**

*Remark*: The proposed Algorithm 1 itself is sufficient to serve as a multi-target state extraction method for the SMC-PHD filter. However, just as the existing measurement-oriented methods, it is limited by the fact that no estimates for undetected targets will be reported. Then Algorithm 2 is proposed to compensate this disadvantage, which is an alternative technique particularly regarding the estimation of undetected targets. It can be combined with Algorithm 1 to further improve the estimation performance in the presence of detection uncertainty. This beneficial result comes with a slight increase in the amount of calculations arising from getting U*_k_* and the clustering method. Fortunately, the clustering method operates only when the missed detections are confirmed by Equation (19). Moreover, the number of particles in U*_k_* is much less than that in the original particle set, which will simplify the clustering process. In terms of computational complexity, the proposed Algorithm 1 has the same complexity of O(*TN*) as the methods in [20,21], where *T* is the number of targets and *N* is the number of particles, while the most popular k-means clustering method has a computational complexity of O(*τTN*), where *τ* is the number of iterations in the clustering procedure.

### 3.3. Notes on Implementation

From an implementation perspective, the proposed Algorithm 1 is potentially engineer-friendly as compared with the popular clustering or clustering-based techniques [15,16,19], where no iterative calculation is involved. Besides, taking advantages of the inherent information in the filtering stage, the variables originally computed in *Step 1* and *Step 2* are exploited to design our method, which makes the method more suitable for the SMC-PHD filter framework.

There are two empirical parameters, *i.e.*, *γ_g_* in Equation (10) and *τ_n_* in Equation (13), which require to be determined when applying the proposed methods. The parameter *γ_g_* is important for eliminating the particles that have negligible normalized likelihood with respect to the collected measurements. At each iteration of the SMC-PHD filter, a specified number of particles per target (denoted by *β*) will be allocated to guarantee a reasonable number of weighted particles for approximating the PHD of individual targets [7], and the updated weight of each particle contributes a certain proportion to the PHD of the underlying target. The normalized likelihood distribution in our method has a similar characteristic with respect to individual particles. It can be interpreted that *γ_g_* is used to select the particles that make certain contributions to the whole distribution in Equation (9) as well as result in non-zero likelihoods corresponding to a given measurement. In general, the particle classification process is sensitive to *γ_g_*. The smaller the *γ_g_*, the more valid particles will be selected for detected targets. Accordingly, some of these particles tend to have negligible updated weights. However, this result will not have an obvious effect on the estimation accuracy for the reason that the weighted mean of the state particles in Algorithm 1 highlights the contributions of the particles with significant weights in each cluster. On the other hand, given an observation model, the value of *γ_g_* highly depends on the ***β*** value used in the filter because all the particles of a target may generate non-zero likelihoods, which hence influence the results of Equation (9). Considering the spatial distribution characteristics of the particles and possible observation uncertainties in practical applications, we choose to use *γ_g_* = 1/*β* as the threshold, which is consistent with the average contribution value of each particle. Equivalently, the contribution degree 1/*β* is acceptable for the valid particles.

In addition, the parameter *τ_n_* in Equation (13) is introduced to give a high confidence level to the measurements which associate with a significant number of particles. Such measurements are referred to as effective measurements and will be useful for state extraction. Like *γ_g_*, *τ_n_* directly determines the number of effective measurements, a proper *τ_n_* can help to eliminate clutter measurements, which in turn allows a high computational efficiency of the proposed method by obviating unnecessary computation due to partitioning particles corresponding to clutter measurements. There is a trade-off between including true measurements and eliminating clutter measurements. When the observation accuracy is high, a relatively large *τ_n_* can be adopted, and *vice versa*. Empirically, 0.1β~0.5β is suggested.

## 4. Simulation

To evaluate the performance of the proposed methods, a two-dimensional tracking scenario with an unknown and time-varying number of targets is considered, where the target dynamic model is exactly the same as that in [7,24]. The position of the sensor platform is assumed to be known at coordinate origin, and the observation equations are given by:

(22) $$\theta_{k}=\arctan\left(\frac{\left[\begin{array}{cccc} 0 & 0 & 1 & 0 \end{array}\right]x_{k}-y_{s}}{\left[\begin{array}{cccc} 1 & 0 & 0 & 0 \end{array}\right]x_{k}-x_{s}}\right)+\varepsilon_{k,1}$$

(23) $$r_{k}=\left\|\left[\begin{array}{cccc} 1 & 0 & 0 & 0\\ 0 & 0 & 1 & 0 \end{array}\right]x_{k}-\left[\begin{array}{l} x_{s}\\ y_{s} \end{array}\right]\right\|+\varepsilon_{k,2}$$

where *ε_k_*_,1_ and *ε_k_*_,1_ are the zero-mean Gaussian white noise with respective standard deviations π/180° and 2 m. Assuming that the survival probability of each target is independent of its state and the value is set to be *p_S,k_* = 0.98 during the simulations. Clutter is uniformly distributed over the region [0, π/2](rad) × [0,700] (m), and the number of clutter measurements per scan is Poisson distributed with a specified mean value *r*. *β* = 500 is used in the SMC-PHD filter, and thus the number of particles varies according to the estimated number of targets. The parameters *γ_g_ =* 1/*β* , *τ_n_* = 100 and λ= 25 (corresponding to *p_g_* = 0.9999) are used for the proposed Algorithms 1 and 2. Besides, the OSPA metric [22] is adopted to evaluate the estimation performance of different methods. The intensity of newborn targets is modelled as [7,15,24]:

(24) $$\gamma_{k}(x)=0.1\sum_{j=1}^{3}N(x;m_{\gamma ,k}^{j},P_{\gamma ,k}^{j})$$

where N(***x***;***m,P***) denotes a normal distribution with mean ***m*** and covariance ***P***, and the values of $\;m_{\gamma ,k}^{j}$ and $P_{\gamma ,k}^{j}$ are configured as $m_{\gamma ,k}^{1}={\left[50,\;7,\;150,\;7\right]}^{T}$, $m_{\gamma ,k}^{2}={\left[355,\;0,\;50,\;7\right]}^{T}$, $m_{\gamma ,k}^{3}={\left[350,\;-7,\;450,\;-7\right]}^{T}$ and $P_{\gamma ,k}^{1}=P_{\gamma ,k}^{2}=P_{\gamma ,k}^{3}=\text{diag}([25,\;10,\;25,\;10])$. We compare the performance of the proposed methods with that of the *k*-means method [15], grouping method [20] and MEAP method [24] via Monte Carlo (MC) simulations.

**Figure 1:**
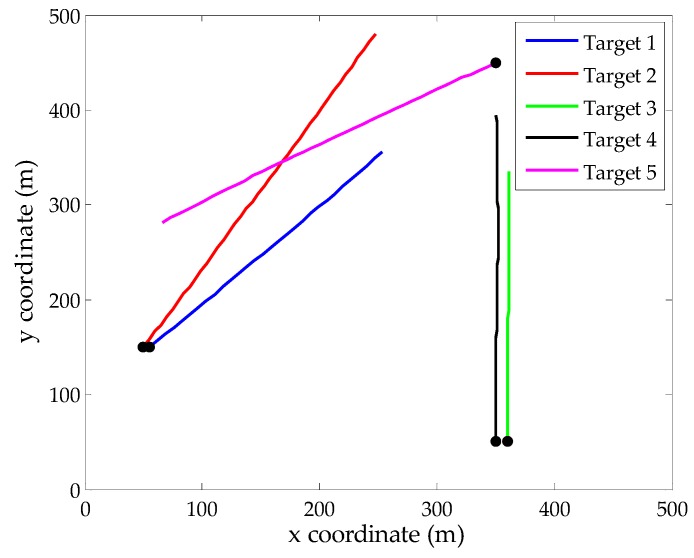
True target tracks in *xy*-plane, the start points for each track are denoted by •.

In the surveillance region, we design a relatively complex multi-target environment with crossing tracks and paralleling motion in close range. More specifically, target 1 exists in the surveillance region from time step 1 to 30. Target 2 and target 5 appear at time step 20 from different positions and their track crossing happens at time step 45. Target 3 and target 4 appear at time step 15 simultaneously and they keep parallel motion until time step 50. Figure 1 shows the true tracks of 5 targets in this scenario. To capture the localization errors of different state extraction methods, the parameters *p* = 2 and *c* = 20 are chosen to generate OSPA metric value.

**Figure 2:**
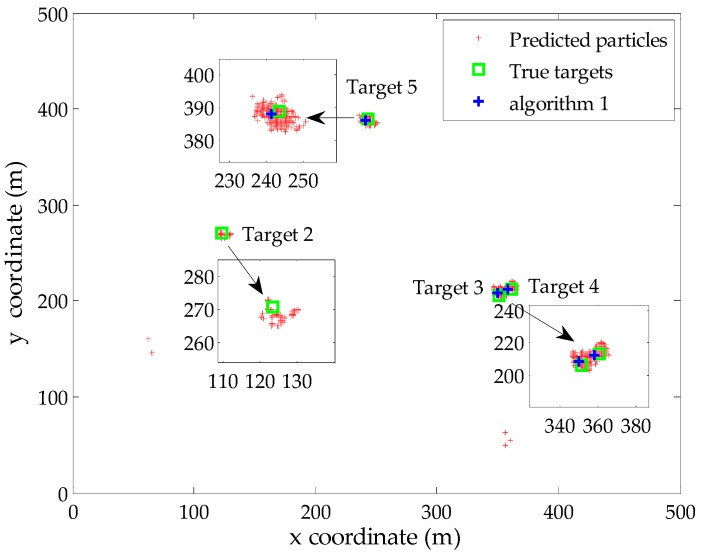
The output of the SMC-PHD filter at time step 35.

To verify the effectiveness of the proposed Algorithms 1 and 2 in an intuitive manner, we present a typical example of the filter output at time step 35 (missed detection occurs). The probability of detection and clutter rate are set to be *p_D_* = 0.90 and *r* = 10 in the simulation, respectively. There exist four true targets during this iteration, while the estimated number of targets is *N*_35_ = 3. Figure 2 gives the global distribution of the predicted particles corresponding to persistent targets at this time step. Besides, the true locations of targets and the extracted locations using Algorithm 1 are also displayed in the same figure. It is clear that the proposed Algorithm 1 provides accurate estimation of the existing three targets based on the estimated number of targets, although the particles of the target 3 and target 4 exhibit significant overlap due to the parallel motion in close space. Besides, target 2 is an undetected target and the existing measurement-oriented methods cannot extract its state in this case, including the proposed Algorithm 1.

**Figure 3:**
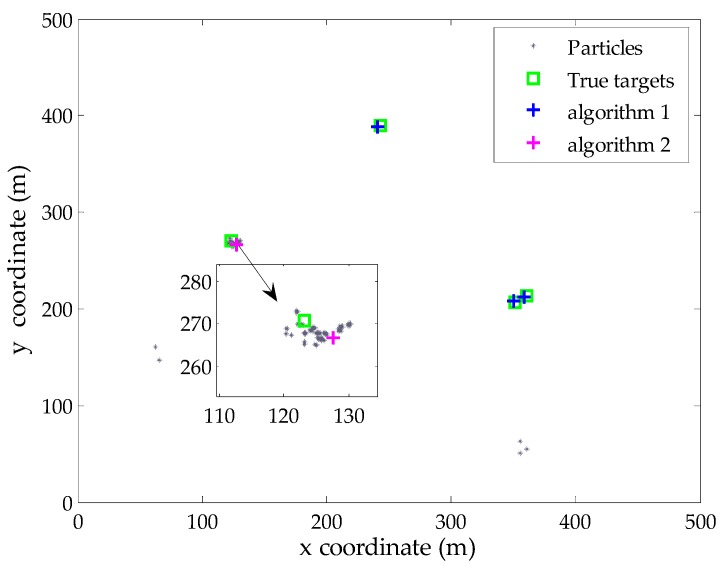
The modified estimate result and selected particles corresponding to undetected target.

Subsequently, the proposed Algorithm 2 is performed. Figure 3 shows the particles (in U*_k_*) corresponding to undetected targets at this time step, as well as the extracted target state from these particles. The results demonstrate that the proposed Algorithm 2 can effectively identify the potential particles of undetected target and then extract the state estimate. As shown in Figure 3, there are a few spurious particles around the birth regions. This phenomenon is caused by the resampling process in the previous iteration, where the filter needs to draw particles from the birth intensity to exploit potential new targets at each scan.

**Figure 4:**
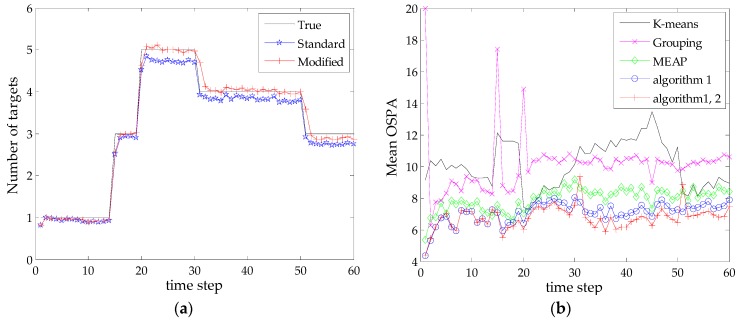
Target number estimate and mean OSPA distance *versus* time (r=10): (**a**) Target number estimate *versus* time; (**b**) Mean OSPA distance *versus* time.

To compare the average performance, 200 MC runs are performed for the SMC-PHD filter with different state extraction methods. The target tracks are fixed but clutter and measurements are independently generated for each trial. Note that the effectiveness of the proposed Algorithm 2 is examined by combining its estimated results with that of the Algorithm 1, namely, the modified results in Equations (20) and (21), in subsequent simulations. Figure 4 shows the statistical results of the estimated number of targets and mean OSPA distances of five methods at each time step. The true number of targets and the estimated results from SMC-PHD filter are shown in Figure 4a. Based on the extracted multi-target state using different methods, the corresponding mean OSPA distances are shown in Figure 4b.

As shown in Figure 4a, the standard SMC-PHD filter gives a satisfactory performance on the target number estimation in this scenario, while the modified estimate by Algorithm 2 follows the true value more closely. In terms of state estimation, it can be seen from Figure 4b that the grouping method shows a slight advantage over the k-means clustering method at most time steps but has a large error when new targets appear. The reason is that the method only reports the estimates whose total weights are above a certain threshold value (we adopt 0.8 as was done in [20]), which is not favorable for the estimation of newborn targets. By contrast, the superiorities of the MEAP method and the proposed methods are remarkable. It can be seen that the proposed Algorithm 1 exhibits a better performance than that of the MEAP method. This can be attributed to the proposed mechanism for selecting particles and measurements, where the contributions of particle likelihoods are evaluated and the scopes of particles are restricted corresponding to effective measurements. Such kind of particles can exactly capture the regions of PHD peaks for state extraction. Moreover, the combination of Algorithms 1 and 2 achieves the best estimation accuracy as compared with all other methods in the presence of detection uncertainty. Figure 4b also indicates that the estimation of undetected targets will cause a short delay response (exhibits large error at time steps 31 and 51) when targets really disappear. In reality, it is difficult to discriminate the two cases in a single iteration.

**Figure 5:**
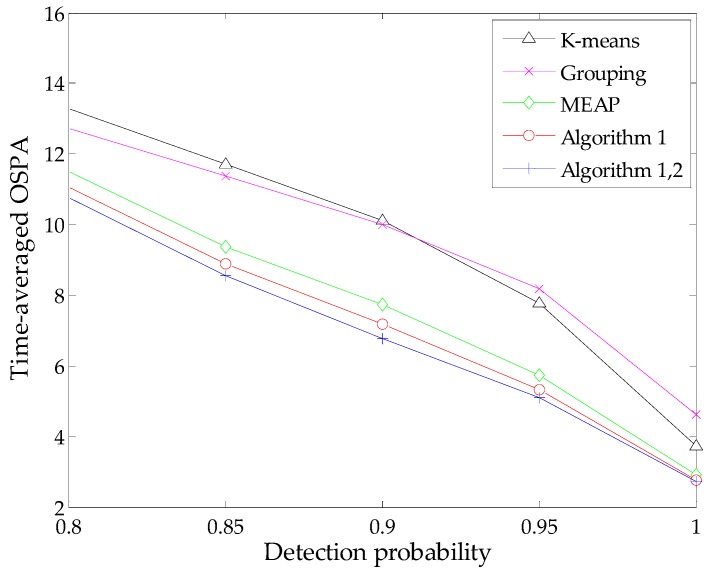
Time-averaged OSPA distance *versus* detection probability (r=10).

**Figure 6:**
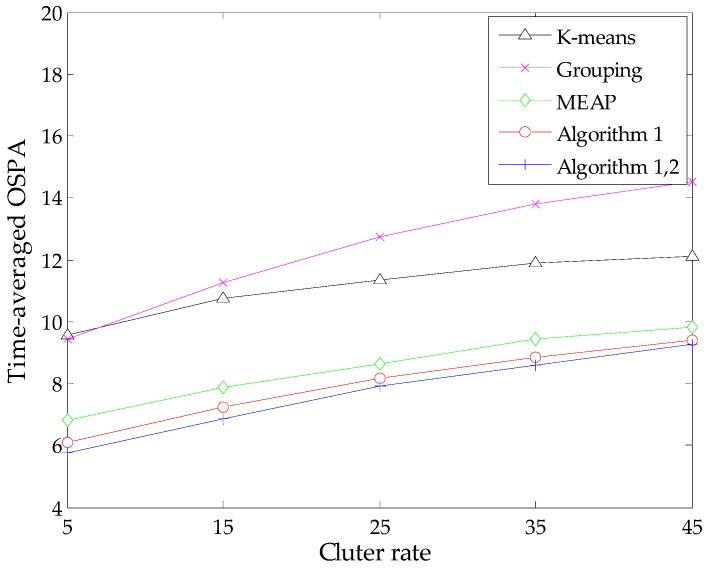
Time-averaged OSPA distance *versus* clutter rate (*p_D_* = 0.90).

To present a more comprehensive evaluation, we also study algorithm performance against different detection probabilities via MC simulations with fixed clutter rate *r* = 10. In addition, similar simulations are performed against various clutter rates under the condition of a constant detection probability *p_D_* = 0.90. We compute the time-averaged OSPA distance of each method during these simulations. The results are presented in Figure 5 and Figure 6, respectively.

As expected, all methods exhibit some performance degradation under the condition of low signal-to-noise ratio. This is because the estimated number of targets given by the SMC-PHD filter becomes increasingly unreliable with an increase of detection uncertainty or the amount of clutters, which in turn has an influence on the state estimation. In addition, when the clutter rate increases, the probability that some clutter measurements appear at the regions of true targets or the birth intensities will increase correspondingly. It is possible that the contributions of these measurements are significant as those of true measurements. Therefore, the measurement-oriented methods (grouping method, MEAP method and Algorithm 1) are prone to be influenced by such clutter measurements. Obviously, the performance degradation of the grouping method is more apparent. Both the results in Figure 5 and Figure 6 demonstrate that the proposed methods yield the best performance in terms of estimation accuracy and robustness. Meanwhile, the improvement of Algorithm 2 seems limited in the case of lower detection uncertainty due to the following reasons: the SMC-PHD filter will lose tracking of targets frequently in such cases, while the estimation of undetected targets is performed under the premise that the corresponding particles exist in the state space. Thus, the improvement tends to be weakened when considering the global effect via evaluating the time-average of the OSPA metric.

## 5. Conclusions

This paper proposes a more practical and effective solution to the problem of extracting multi-target states from the SMC-PHD filter. Based on the proposed measurement and particle validation mechanism, the estimates of detected targets and undetected targets are extracted separately within the filter framework. For the detected targets, the estimation is achieved by partitioning particles with respect to effective measurements according to their association probabilities, which is more computationally efficient than the traditional clustering-based algorithms. For the undetected targets, we first identify the corresponding particles and then extract the estimates by exploiting clustering method. Simulation results demonstrate that the proposed method outperforms the state-of-the-art measurement-oriented methods and the popular k-means clustering method in terms of both estimation accuracy and reliability in the presence of clutter and detection uncertainty. Moreover, it is possible to extend the proposed methods to SMC implementation of the cardinalised PHD filter [12]. However, like most of the existing solutions, the proposed methods are also heuristic to some extent, and there exist empirical parameters during the implementation of our methods. In the further work, developing state extraction method with strong theoretical justification for the PHD-based filter is an important topic.

## Abbreviations

The following abbreviations are used in this manuscript:

FMM	Finite mixture models
JPDA	Joint probabilistic data association
MEAP	Multi-expected a posterior
MHT	Multiple hypothesis tracking
MTT	Multi-target tracking
OSPA	Optimal Sub-pattern Assignment
PHD	Probability hypothesis density
RFS	Random finite sets
SMC	Sequential Monte Carlo

## References

[1] Shalom Y.B., Daum F., Huang J. (2009). The probabilistic data association filter estimation in the presence of measurement uncertainty. IEEE Control Syst. Mag..

[2] Blackman S.S. (2004). Multiple hypothesis tracking for multiple target tracking. IEEE Aerosp. Electron. Syst. Mag..

[3] Habtemariam B., Tharmarasa R., Thayaparan T., Mallick M., Kirubarajan T. (2013). A multiple-detection joint probabilistic data association filter. IEEE J. Sel. Top. Signal Process..

[4] Mahler R. (2003). Multitarget Bayes filtering via first-order multitarget moments. IEEE Trans. Aerosp. Electron. Syst..

[5] Mahler R. (2007). PHD filters of higher order in target number. IEEE Trans. Aerosp. Electron. Syst..

[6] Vo B.N., Vo B.T., Phung D. (2014). Labeled random finite sets and the Bayes multi-target tracking filter. IEEE Trans. Signal Process..

[7] Vo B.N., Singh S., Doucet A. (2005). Sequential Monte Carlo methods for multi-target filtering with random finite sets. IEEE Trans. Aerosp. Electron. Syst..

[8] Whiteley N., Singh S., Godsill S. (2010). Auxiliary particle implementation of probability hypothesis density filter. IEEE Trans. Aerosp. Electron. Syst..

[9] Vo B.N., Ma W.K. (2006). The Gaussian mixture probability hypothesis density filter. IEEE Trans. Signal Process..

[10] Yoon J.H., Kim D.Y., Yoon K.J. (2012). Efficient importance sampling function design for sequential Monte Carlo PHD filter. Signal Process..

[11] Zhang F.H., Buckl C., Knoll A. (2014). Multiple Vehicle Cooperative Localization with Spatial Registration Based on a Probability Hypothesis Density Filter. Sensors.

[12] Ristic B., Clark D., Vo B.N., Vo B.T. (2012). Adaptive target birth intensity in PHD and CPHD filters. IEEE Trans. Aerosp. Electron. Syst..

[13] Battistelli G., Chisci L., Morrocchi S., Papi F., Farina A., Graziano A. (2012). Robust multisensor multitarget tracker with application to passive multistatic radar tracking. IEEE Trans. Aerosp. Electron. Syst..

[14] Ristic B. Efficient update of persistent particles in the SMC-PHD filter. Proceedings of the International Conference on Acoustics, Speech and Signal Processing.

[15] Clark D.E., Bell J. (2007). Multi-target state estimation and track continuity for the particle PHD filter. IEEE Trans. Aerosp. Electron. Syst..

[16] Dunne D., Tharmarasa R., Lang T., Kirubarajan T. SMC-PHD-based multi-target tracking with reduced peak extraction. Proceedings of the SPIE 7445, Signal and Data Processing of Small Targets.

[17] Liu W.F., Han C.Z., Lian F., Zhu H.Y. (2010). Multitarget state extraction for the PHD filter using MCMC approach. IEEE Trans. Aerosp. Electron. Syst..

[18] Tobias M., Lanterman A.D. (2008). Techniques for birth-particle placement in the probability hypothesis density particle filter applied to passive radar. IET Radar Sonar Navig..

[19] Tang X., Wei P. (2011). Multi-target state extraction for the particle probability hypothesis density filter. IET Radar Sonar Navig..

[20] Ristic B., Clark D., Vo B.N. Improved SMC implementation of the PHD filter. Proceedings of the 13th International Conference on Information Fusion.

[21] Lin L.K., Xu H., Sheng W.D., Wei A. (2012). Multi-target state-estimation technique for the particle probability hypothesis density filter. Sci. China Inform. Sci..

[22] Schuhmacher D., Vo B.T., Vo B.N. (2008). A consistent metric for performance evaluation of multi-object filters. IEEE Trans. Signal Process..

[23] Baum M., Willett P., Hanebeck U.D. MMOSPA-based track extraction in the PHD filter-a justification for *k*-means clustering. Proceedings of the 53th IEEE Conference on Decision and Control.

[24] Li T.C., Sun S.D., Bolić M., Corchado J.M. (2016). Algorithm design for parallel implementation of the SMC-PHD filter. Signal Process..

[25] Pierre D.M., Houssineau J., Peters G.W., Matsui T. (2015). Particle Association Measures and Multiple Target Tracking. Theoretical Aspects of Spatial-Temporal Modeling.

[26] Erdinc O., Willett P., Bar-Shalom Y. (2009). The bin-occupancy filter and its connection to the PHD filters. IEEE Trans. Signal Process..

[27] Blackrnan S., House A. (1999). Design and Analysis of Modern Tracking Systems.

